# WNK1, a molecular crowding sensor, links phase separation to cellular physiological stress

**DOI:** 10.1002/mco2.232

**Published:** 2023-03-18

**Authors:** Tinghui Lan, Fangfang Zhou, Long Zhang

**Affiliations:** ^1^ Institutes of Biology and Medical Science Soochow University Suzhou China; ^2^ MOE Laboratory of Biosystems Homeostasis and Protection and Innovation Center for Cell Signaling Network Life Sciences Institute Zhejiang University Hangzhou China

1

A recently published study in Cell by Boyd‐Shiwarski et al.^1^ revealed that WNK kinases sense intracellular molecular crowding and restore cell volume via phase separation (PS). Cells are suffer from hyperosmotic stress, which leads to volume shrinkage and a decrease in intracellular space, resulting in molecular crowding (Figure 1A). WNK1 rapidly senses molecular crowding, triggers PS via the C‐terminal domain (CTD), and recruits downstream effectors to form condensates. The SPAK and OSR1 effector kinases are activated in condensates and transported to the cytosol to regulate the NKCC1 and KCC ion channels. Eventually, resulting in the obligate reclamation of water and cell volume is recovered, this rapid response termed regulatory volume increase1 (RVI) (Figure 1B).

**FIGURE 1:**
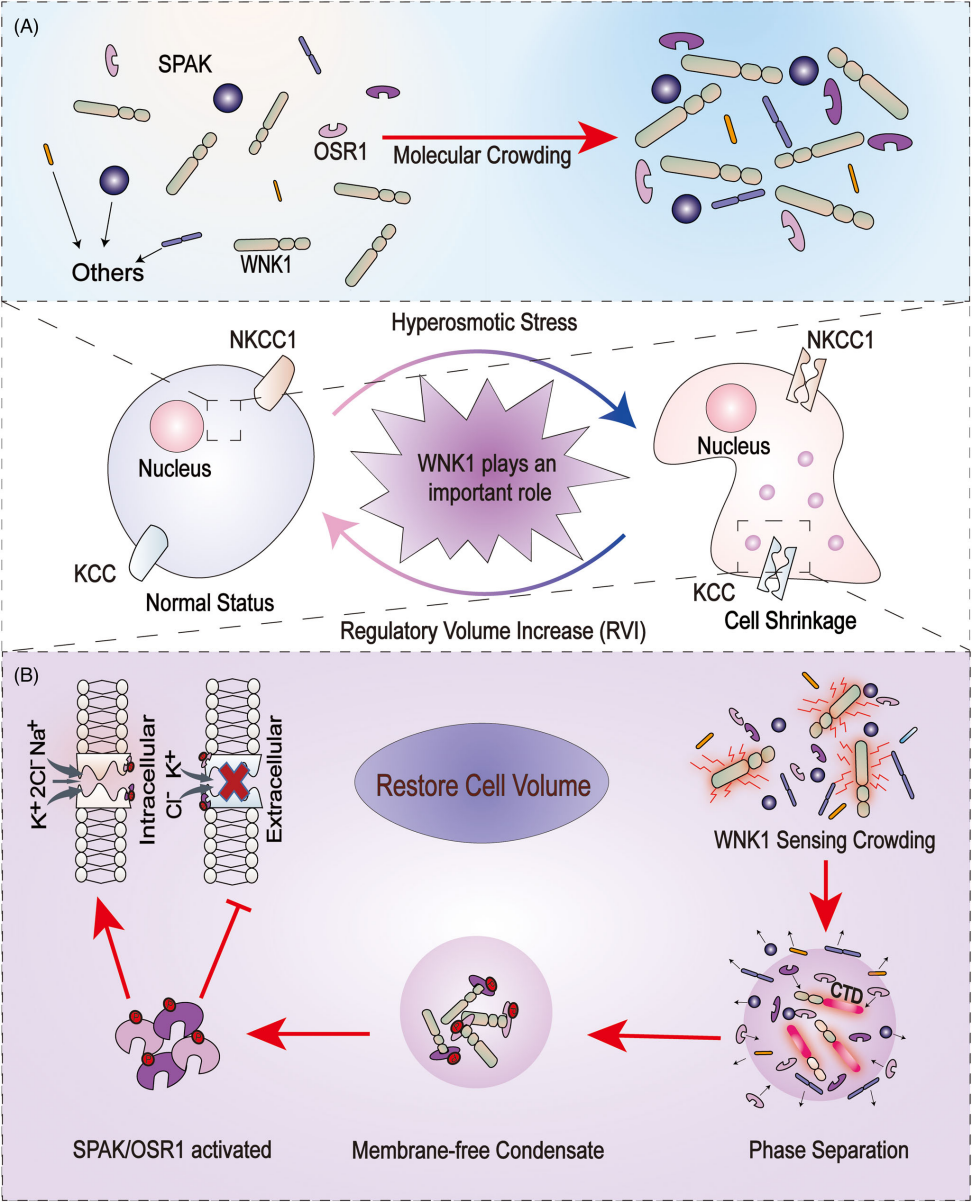
WNK1 senses molecular crowding and restores cell volume. (A) Hyperosmotic stress‐induced cell shrinkage. (B) WNK is activated in response to molecular crowding due to hyperosmotic stress‐induced cell shrinkage. Following that, the WNK1 C‐terminal domain (CTD) mediates phase separation (PS) to form condensates. WNK1 phosphorylates SPAK/OSR1, which activates SPAK/OSR1 in condensates. Finally, SPAK/OSR1 regulates NKCC1/KCC ion channels, trigger regulatory volume increase (RVI), and restore cell volume.

PS can be triggered by various cellular stresses, including temperature, pH, and hyperosmolarity.^2^ Meanwhile, PS can produce membrane‐free biomolecular condensates, in which specific biomolecules are concentrated and serve biological functions.^3^ For instance, the phosphorylation of the Alzheimer's disease‐related protein tau by cyclin‐dependent kinase 2 is accelerated in condensates,^4^ and biomolecular condensates are implicated in the regulation of postsynaptic density at neuronal synapses in synaptic signaling and plasticity.^3^ Numerous studies have demonstrated the significance of biomolecular condensates in physiology and pathophysiology.^2^, ^3^ In addition to triggering PS, hyperosmotic stress can also activate WNK1.^5^ WNK1 belongs to the with‐no‐lysine (K) protein kinase family and is associated with Cl^−^, K^+^, and Na^+^ ion transport. When cells are subjected to hyperosmotic stress, WNK1 is activated, mediates ion flow, and triggers RVI to prevent cell death and triggers RVI to prevent cell death. When WNK1 is activated by hyperosmotic stress, it colocalizes with downstream effector kinases in cytoplasmic puncta, when these puncta were first described in 2007, it was assumed that these puncta were membrane‐bound intracellular vesicles; however, recent work by Boyd‐Shiwarski has characterized these WNK1 puncta as membrane‐less. This is consistent with other proteins that undergo PS and form membrane‐less compartments.^5^ However, PS triggered by molecular crowding caused by hyperosmotic stress appears to be a common mechanism by which multimeric proteomes exert biological functions.^3^

Boyd‐Shiwarski et al.^1^ are the first to report how molecular crowding causes WNK1 to phase separate into cytoplasmic puncta, and these findings have major implications for the physiologic regulation of RVI. Surprisingly, the formation of WNK1 cytoplasmic puncta was found to be dependent on the degree of hyperosmotic stress, and electron hyperdensities observed under correlative light and electron microscopy experiments were typical of condensates formed through PS. These findings suggest that WNK1 cytoplasmic puncta were not vesicular membranes but rather membraneless condensates formed by PS triggered by hyperosmotic stress. Meanwhile, the authors demonstrated that the WNK1 CTD is critical in mediating WNK1 PS and condensate formation. Interestingly, the authors found that, while C‐terminal coiled‐coil domains (CT CCDs) are not required for PS, the presence of CT CCDs enable WNK1 condensates to form at the appropriate time.

Second, the authors conducted a bioinformatics analysis to determine the WNK CTD amino acid composition during the evolution from protists to humans. Although the primary amino acid composition of the WNK CTD differs between vertebrates and invertebrates, both mediate condensate formation in a similar manner. Then, studies on the WNK1 homolog *Drosophila melanogaster* WNK (Dm WNK) revealed that, similar to mammalian WNK1, Dm WNK CTD also generates condensates in response to hyperosmotic stress. These findings suggest that, while the amino acid compositions of vertebrate and invertebrate WNK CTDs are not identical, the ability of the WNK CTD to mediate PS to generate condensates is evolutionarily conserved.

Subsequently, Boyd‐Shiwarski et al.^1^ demonstrated that WNK1 CTD is required for RVI. In addition, the influx of the K^+^ congener rubidium (Rb^+^) was studied under isotonic and hypertonic conditions in WNK1/WNK3 double knockout cells expressing full‐length or the 1–494 WNK1 fragment. The authors demonstrated that WNK1 CTD plays an important role in NKCC1 transport. Another interesting finding is that after introducing a sequence capable of causing PS to replace the original CTD sequence of WNK1, the WNK1 kinase domain can still phosphorylate downstream effector kinases, thereby mediating RVI.

Finally, Boyd‐Shiwarski et al.^1^ utilized the molecular crowding agent Ficoll, which can trigger the protein PS in vitro, to investigate the effect of molecular crowding on WNK1 condensate formation. Following intracytoplasmic microinjection of Ficoll, the degree of molecular crowding in the cytoplasm increased, and WNK1 condensate formed. In addition, when WT HEK293T cells transiently expressing either full‐length WNK1 or an N‐terminal fragment of WNK1 (1‐494), only the full‐length WNK1 expressing cells exhibited PS. This suggests that WNK1 is an intracellular molecular crowding sensor that triggers PS to regulate cell volume.

Boyd‐Shiwarski et al.^1^ first demonstrated that cytoplasmic puncta are membraneless condensates formed by hyperosmotic stress‐induced PS via WNK1. Meanwhile, the authors found that WNK1 responds to hyperosmotic stress by sensing molecular crowding and that its CTD is indispensable in the process of condensate formation. Furthermore, the WNK1 CTD is evolutionarily conserved, suggesting that PS may be fundamental cell behavior. The authors demonstrated the significance of protein phase behavior in cellular physiological processes, which was previously believed to correlate little with physiological relevance. In addition, the authors refuted the notion that WNK1 cytoplasmic puncta are membrane vesicles, which has significant implications for further studies of the dynamics of WNK1 condensates and whether additional regulatory mechanisms exist in the condensates.

Several issues remain to be resolved in future research. For instance, previous studies have shown that phosphorylation of different WNK1 amino acid residues can activate or inhibit its activity during hyperosmotic stress.^5^ However, there is no direct evidence for the involvement of other proteins in this process. It will be critical to elucidate the underlying mechanism of WNK1 phosphorylation in condensates. This will not only be beneficial for exploring relevant physiological regulatory mechanisms but also be important for the development of WNK1‐SPAK/OSR1 pathway targeted‐drugs. In addition, the transport mechanism of SPAK/OSR1 following phosphorylation in the cytosol is also worth investigating, which will facilitate the discovery of relevant transporters as well as regulatory targets.

In summary, the authors established for the first time a direct link between WNK1‐mediated PS and physiological stress responses, which is critical for future research into the relationship between PS and cell physiology.

## AUTHOR CONTRIBUTIONS

Tinghui Lan performed the drawing of the images and the writing of the article. Fangfang Zhou and Long Zhang provided modifications. All authors have read and approved the final manuscript.

## CONFLICT OF INTEREST STATEMENT

The authors declare no competing interests.

## ETHICS STATEMENT

No ethical approval was necessary for this work.

## Data Availability

No.
